# Revisiting vocal perception in non-human animals: a review of vowel discrimination, speaker voice recognition, and speaker normalization

**DOI:** 10.3389/fpsyg.2014.01543

**Published:** 2015-01-13

**Authors:** Buddhamas Kriengwatana, Paola Escudero, Carel ten Cate

**Affiliations:** ^1^Behavioural Biology, Institute for Biology Leiden, Leiden UniversityLeiden, Netherlands; ^2^Leiden Institute for Brain and Cognition, Leiden UniversityLeiden, Netherlands; ^3^The MARCS Institute, University of Western SydneySydney, NSW, Australia

**Keywords:** asymmetries in vowel perception, comparative cognition, general auditory approach, voice perception, language evolution, animal behavior, speaker normalization

## Abstract

The extent to which human speech perception evolved by taking advantage of predispositions and pre-existing features of vertebrate auditory and cognitive systems remains a central question in the evolution of speech. This paper reviews asymmetries in vowel perception, speaker voice recognition, and speaker normalization in non-human animals – topics that have not been thoroughly discussed in relation to the abilities of non-human animals, but are nonetheless important aspects of vocal perception. Throughout this paper we demonstrate that addressing these issues in non-human animals is relevant and worthwhile because many non-human animals must deal with similar issues in their natural environment. That is, they must also discriminate between similar-sounding vocalizations, determine signaler identity from vocalizations, and resolve signaler-dependent variation in vocalizations from conspecifics. Overall, we find that, although plausible, the current evidence is insufficiently strong to conclude that directional asymmetries in vowel perception are specific to humans, or that non-human animals can use voice characteristics to recognize human individuals. However, we do find some indication that non-human animals can normalize speaker differences. Accordingly, we identify avenues for future research that would greatly improve and advance our understanding of these topics.

## INTRODUCTION

The answer to how humans perceive speech has eluded researchers for over half a century (Jusczyk and Luce, 2002; Samuel, 2011). Remarkably, studies in non-human animals (hereafter referred to as animals) have shown that animals can also solve certain problems that are crucial to human speech perception, such as lack of invariance and compensation for co-articulation (e.g., Kluender et al., 1987; Lotto et al., 1997a). The results of these pioneering studies have shown remarkable similarities, but also differences, in perception, discrimination, and sensitivity to acoustic properties of speech in humans and animals (reviewed by Kuhl, 1981; Lotto et al., 1997b; Kluender et al., 2005; Beckers, 2011; Carbonell and Lotto, 2014; ten Cate, 2014). Consequently, many researchers have adopted the general auditory approach outlined by Diehl et al. (2004), which is a framework for the idea that human speech perception is achieved via general learning mechanisms and auditory principles common to humans and animals.

From a general auditory approach, categorical perception occurs at natural psychophysical boundaries constrained by the functioning of the auditory system (Kuhl and Miller, 1975), compensation for coarticulation is possible by contrasting spectral patterns of high and low energy in particular frequency regions (Lotto et al., 1997b; Diehl et al., 2004), and the lack of invariance in speech can be solved in ways similar to concept formation for visual categories that cannot be defined by any single cue (Kluender et al., 1987). Furthermore, phonetic category learning by humans and animals can potentially be achieved via statistical learning and perceptual learning mechanisms. Statistical learning can account for how human infants (Maye et al., 2002), but also rats (Pons, 2006), use the distributional properties of acoustic input to learn phonetic categories, such that exposure to a speech sound continuum with unimodal or bimodal distribution can result in acquisition of one or two phonetic categories, respectively. Statistical learning also appears to underlie our ability to use recurring sound sequences in speech to denote word boundaries (Saffran et al., 1996; Pelucchi et al., 2009), which is also observed when stimuli are tones or musical sounds (Saffran et al., 1999; Gebhart et al., 2009). Thus, statistical learning is a general learning mechanism that is not specific to speech, but is useful for speech perception because it can be used to map acoustic properties onto phonetic categories in a probabilistic manner (Holt et al., 1998).

Altogether, these studies culminate to form the current dominant view that at least several processes involved in speech perception in humans can be traced back to predispositions, learning mechanisms and rudimentary features of vertebrate cognitive and auditory systems also present in other species (e.g., Carbonell and Lotto, 2014). Nevertheless, an enduring and central question in the evolution of speech and language is whether our extraordinary abilities to deal with the enormous variety of speech sound and voices is a matter of degree compared to the abilities of animals, or the result of an evolutionary quantum leap resulting in novel and unique specialized mechanisms. Animals have, of course, never been under selection to process speech sounds or recognize voices. If they would also process their own communication sounds similar to how humans do, this might indicate a more general mechanism, but might also indicate an independently evolved perceptual mechanism highly specific to their own vocal communication. Hence, a much stronger indication for the presence of general perceptual mechanisms would be if animals process human speech sounds similar to humans. This would indicate that our ability to handle the complexities of speech likely arose from an amalgamation and adaptation of simpler mechanisms present in other vocalizing animals. Comparative studies can thus provide an invaluable window into the uniqueness and origin of speech processing mechanisms.

The objective of this paper is to extend the discussion of species-shared perceptual mechanisms to aspects of human speech perception and voice recognition that have not been considered central to conventional theories of speech perception, but nonetheless cannot be ignored. Specifically, we focus our review on asymmetries in vowel perception, speaker voice recognition, and speaker normalization and underline how these areas of research can benefit from incorporating comparative perspectives. Reviewing these topics contributes significantly to the debate about the nature and specialness (or generality) of human speech perception because asymmetries in vowel perception may play a crucial role in the development of infant speech perception (Polka and Bohn, 2011), voice perception impacts speech perception (e.g., Nygaard and Pisoni, 1998), and intrinsic speaker normalization is just as meaningful an issue as extrinsic speaker normalization because both are concerned with the problem of perceptual invariance in vowel perception (e.g., Johnson, 2005). Thus, our manuscript provides the first detailed review necessary for a more thorough understanding of whether the mechanisms that mediate asymmetries in vowel perception, voice recognition, and speaker normalization in humans arose from an evolutionary quantum leap, or from tuning and remodeling of existing mechanisms that are also present in non-human species (whether by common descent or by independent evolution unconnected to the presence of speech).

## ASYMMETRIES IN VOWEL PERCEPTION

Polka and Bohn (2003, 2011) review numerous studies on vowel discrimination in human infants and adults. These studies demonstrate a striking directional asymmetry: discrimination of native or non-native vowels by infants and of non-native vowels by adults is easier when the change is from a vowel occupying a more central position in the F1/F2 vowel space to a vowel occupying a more peripheral position (e.g., from /e/ to /i/). Of the dozens of studies reviewed, only one showed that a change from a more to a less peripheral vowel was easier to discriminate than a change in the reverse direction (Best and Faber, 2000 as cited in Polka and Bohn, 2003, 2011), and this was attributed to effects of vowel rounding in F3. Polka and Bohn (2003, 2011) assert that this directional asymmetry helps infants to acquire phonetic categories because the most peripheral vowels /i/, /a/, and /u/ found in all human languages act as stable referents from which infants can perceptually organize their vowel space (see, Polka and Bohn, 2011 for these ideas in the context of the natural referent vowel framework). The authors propose that these biases are specific to humans due to their role in organizing the vowel space and presence very early in development. Emphatically, Polka and Bohn (2003, 2011) claim that these directional asymmetries do not reflect a general auditory processing bias and therefore will not be present in other animals.

To support their claim of species-specificity in the asymmetries observed in human vowel perception, Polka and Bohn (2003) examined vowel discrimination data from red-winged blackbirds (*Agelaius phoeniceus*), pigeons (*Columba livia*), and cats (Hienz et al., 1981, 1996). While red-winged blackbirds and cats (but not pigeons) also exhibited asymmetries in vowel perception, discrimination was almost always easier when formant frequencies were shifted upward (e.g., from /ɔ/ to /ɑ/ or from /ʊ/ to /æ/).

These results, however, do not resolutely show that the central to peripheral bias found in humans is uniquely human. This is because these experiments do not test discrimination between more and less peripheral vowels. Hienz et al. (1981) tested discrimination of the peripheral vowels /ɔ/, /ɑ/, /æ/, /ε/, which does not test whether animals find the change from a central vowel to a peripheral vowel easier to discriminate. Hienz et al. (1996) tested discrimination between the slightly more central vowel /ʊ/ and the peripheral vowels /ɑ/, /æ/, and /ε/, and found that the change from /ʊ/ to /ɑ/, /ʊ/ to /æ/, and //ʊ/ to /ε/ was easier to discriminate than the change in the opposite direction (e.g., /ɑ/ to /ʊ/). Therefore, these results in fact suggest that animals also find the change from a central vowel to peripheral vowel easier to discriminate. Nevertheless, differences in discrimination by humans and non-human animals on the same speech contrasts are certainly needed to conclude that the asymmetry patterns observed in humans are truly unique to humans. The only contrast that was tested in both humans and red-winged blackbirds was the /ε/-/æ/ contrast. In this case, 6–8 and 10–12 month-old human infants found the discrimination easier if the change occurred from /ε/ to /æ/, whereas birds found the change in the reverse direction easier to discriminate (Hienz et al., 1981; Polka and Bohn, 1996). This directional asymmetry was recently found in even younger infants (2–3 month-olds) that were exposed to /ε/ and /æ/ vowels that were bimodally distributed along an [ε-æ] continuum (Wanrooij et al., 2014), and was treated as further evidence to support the central to peripheral bias in humans (Bohn and Polka, 2014). However, as both /ε/ and /æ/ occupy peripheral positions in vowel space, this asymmetry can only falsify the central to peripheral bias if we assume that the change from /ε/ to /æ/ is easier to discriminate because /æ/ is closer to the vowel referent /a/ than /ε/ is to the vowel referent /i/ – according to the natural vowel referent framework, /a/, /i/, and /u/ are vowel referents because they are the most peripheral vowels (Polka and Bohn, 2011). Whether this assumption is correct is not explicitly stated in Polka and Bohn (2003, 2011), but if it is then it is the only vowel contrast so far that could be argued to reflect a human-specific vowel discrimination bias.

**FIGURE 1:**
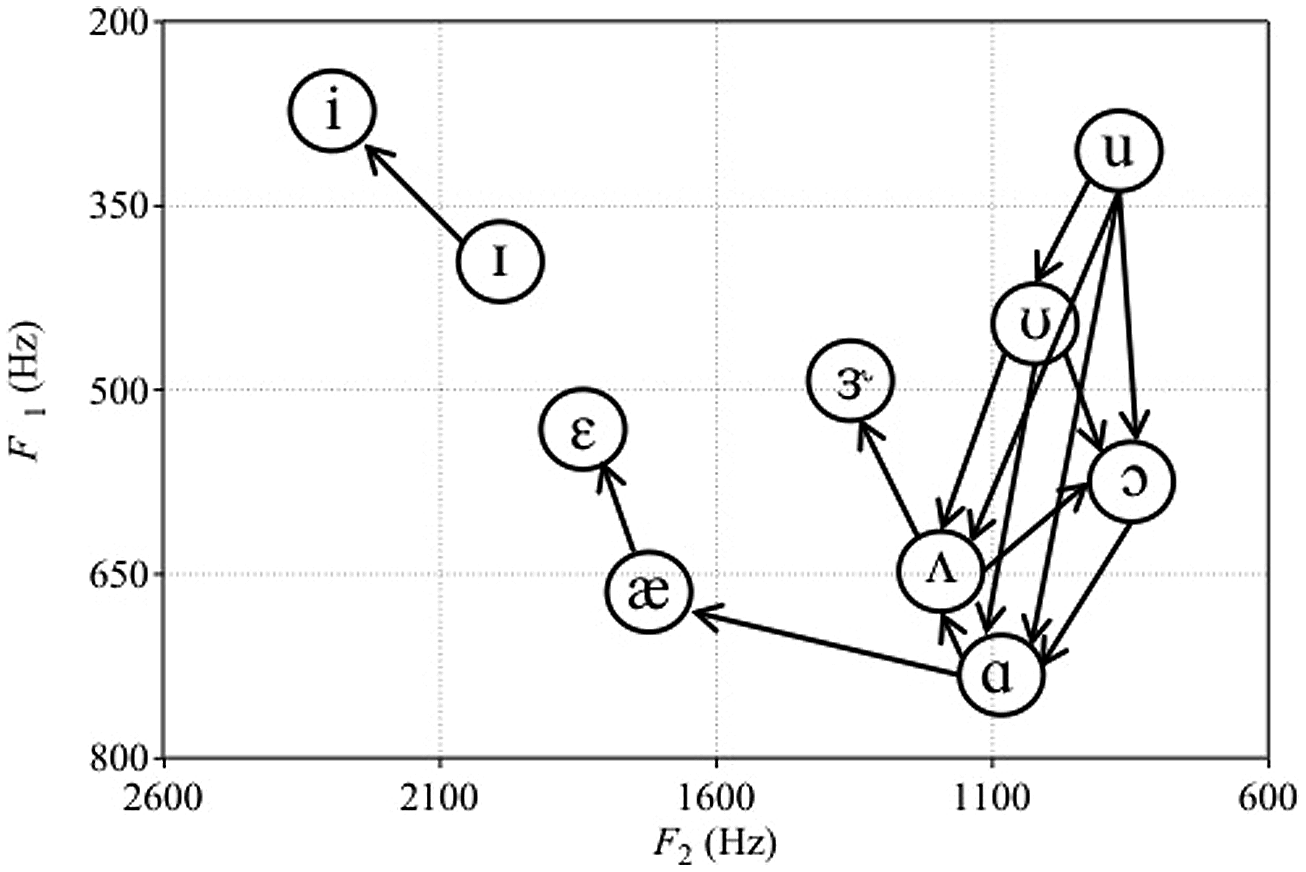
**Plot of the asymmetries in vowel discrimination of vervets from Sinnott (1989).** Arrows represent the direction of change that was easier to discriminate. For macaques, only the ɑs-ʌs contrast was easier to discriminate than the ʌs-ɑs contrast. F1 and F2 values of vowel tokens are those reported in Sinnott (1989).

Missing from Polka and Bohn’s (2003, 2011) reviews was work by Sinnott (1989), who compared detection and discrimination of synthetic English vowels by human adults and monkeys (vervets *Cercopithecus aethiops* and Japanese macaque *Macaca fuscata*). Subjects had to discriminate between two sets of vowels: /i-ɪ-ε-æ-ɝ-ʌ-ɑ/ (mostly front vowels with higher F2) and /ʌ-ɑ-ɔ-ʊ-u/ (mostly back vowels with lower F2). Sinnott (1989) used a repeating standard XA task, where subjects pressed a lever to hear a repeating standard vowel followed by two repetitions of a comparison vowel. Subjects had to release the lever if they perceived the comparison vowel to be different from the standard vowel. Every vowel in each of the two sets served as both standard and comparison vowels. We used these data to assess the asymmetries in monkey vowel discrimination (Sinnott, 1989, Table II, p. 560). These directional asymmetries are portrayed visually in **Figure 1**. We only considered directional asymmetries to be present when the difference between the percent of time a vowel comparison was missed following a particular standard was greater than 25. For example, the /u-ʊ/ asymmetry for vervets was included because vervets missed the change from /u/ to /ʊ/ 93 percent of the time, but missed the change from /ʊ/ to /u/ only 2% of the time.

**Figure 1** shows that, for back vowels, vervets perform similarly to red-winged blackbirds and cats (Hienz et al., 1981, 1996). That is, their discrimination of back vowels is enhanced if the F1 and F2 of the comparison vowel are increased relative to the standard vowel. However, vervets and macaques (but not adult humans) most likely perceive decreases in F2 of these vowels as decreases in intensity (Sinnott, 1989), and earlier work by Sinnott et al. (1985) showed that vervets and macaques have difficulty detecting decrements in intensity. That is, humans, macaques, and vervets required similar intensities to be able to detect front vowels, but monkeys required back vowels to be presented ∼10–20 dB louder than humans to be able to detect them (Sinnott, 1989). Examination of F1, F2, and F3 values of the vowel stimuli showed that monkeys’ detection thresholds were correlated with decreases in F2. This suggested that monkeys required higher intensities in order to be able to detect vowels that decrease in F2 (Sinnott, 1989). Decreased sensitivity to pure tones at lower frequencies of less than 1.0 kHz has also been reported in monkeys compared to humans (Owren et al., 1988). Consequently, perceptual asymmetries involving back vowel contrasts may simply reflect the difficulty that monkeys have if the decrease in F2 from the standard to comparison vowel is perceived as a decrease in intensity. When vowel detection thresholds of humans and monkeys are most similar (i.e., for the front vowels /i/ and /ɪ/ Sinnott, 1989), vervets show the same directional asymmetry as human infants (Swoboda et al., 1978; Dejardins and Trainor, 1998). Therefore, we might expect infants to show a similar directional asymmetry when tested on low back contrasts if they also perceive decreases in F2 as decreases in vowel intensity. This is because infants, like monkeys, are unable to discriminate stimuli that decrease in intensity (Sinnott and Aslin, 1985).

Therefore, to demonstrate that the directional asymmetries proposed by Polka and Bohn’s (2011) natural referent vowel framework are exclusive to humans, we see an absolute need for more studies on humans and animals that use identical stimuli and comparable experimental designs in order to enable between-species comparisons of asymmetries in vowel perception. In particular, we encourage studies in human infants that test for perceptual asymmetries between low back contrasts that have already been examined in animals, such has the /ʊ/ – /ɑ/ contrast (where cats, red-winged blackbirds, and monkeys find the change from /ʊ/ to /ɑ/ easier than the change from /ɑ/ to /ʊ/), and the /u/ – /ʊ/ and /ɑ/ – /ʌ/ contrasts (where monkeys’ performances contradict the predictions of the central-to-peripheral asymmetry hypothesis). Lastly, to properly delineate the function of perceptual asymmetries in vowel perception in humans, we must also understand the causes and possible functions of directional asymmetries in auditory perception in animals. For example, do animals exhibit directional asymmetries in detecting a change in their species-specific communication, and do they make use of these perceptual asymmetries? Or are directional asymmetries a by-product of properties of auditory systems (and thus have no functional use)?

Regardless of whether directional biases in vowel perception are found to be uniquely human, general perceptual biases in human audition may further our understanding of the existence of vowel asymmetries. Various auditory perceptual biases have been found, such as sounds with increasing intensity being perceived as closer than sounds than sounds with decreasing intensity (Neuhoff, 1998), ramped sounds being perceived as having greater intensity and longer duration than damped sounds (Irino and Patterson, 1996; Schlauch et al., 2001), and frequency modulated sounds being easier to detect among pure tones distractors than the reverse (Cusack and Carlyon, 2003). Some of these asymmetries appear to have plausible evolutionary explanations. For instance, rising harmonic sound intensities are reliable indicators of an approaching sound source, thus humans and monkeys perceive the source as being closer than reality when sound intensity increases (but not decreases) in order to account for a “margin of safety” (Neuhoff, 1998; Ghazanfar et al., 2002).

## SPEAKER VOICE RECOGNITION

The speech signal not only contains linguistic information, but also nonlinguistic information about the speaker from his/her voice, such as age, gender, and socio-linguistic background. Voices are sounds generated by vibrations of the vocal folds in the larynx, which are then modified by the vocal tract, leading to an enhancement of particular frequencies (i.e., formant frequencies). The fundamental frequency and formant frequencies in a person’s voice are influenced by the size of their vocal folds and vocal tract, which is why men, with their larger vocal folds and vocal tract, tend to have lower fundamental frequencies and formant frequencies than women and children (reviewed in Belin et al., 2004; Latinus and Belin, 2011). Voice differences between speakers may be related to slight variations in the vocal apparatus of different individuals. Differences in the way the vocal apparatus is used, either intentionally or inadvertently, contributes to the distinctiveness of voices. For example, nasal voices are produced when the velum is in a slightly lowered position and breathy voices are produced when the vocal folds remain slightly parted during speaking (see Story and Titze, 2002). Human adults can reliably detect a speaker’s approximate age, gender, and accented speech (Hartman, 1979; Flege, 1984; Mullennix et al., 1995; Bachorowski and Owren, 1999), and can also identify familiar speakers from hearing their voices (Bricker and Pruzansky, 1976). Importantly, speaker voice characteristics interact with linguistic comprehension during speech perception: familiar speakers elicit better performance in a word identification task than unfamiliar speakers (Nygaard and Pisoni, 1998), and vowel, word, and consonant-vowel identification are more accurate in single speaker compared to multiple speaker conditions (Strange et al., 1976, 1983; Assmann et al., 1982; Magnuson and Nusbaum, 2007). Knowledge of a speaker’s accent and gender also increases word and vowel identification accuracy, respectively (Johnson, 2005; Trude and Brown-Schmidt, 2012; Smith, 2014). Consequently, a complete account of how humans perceive speech also requires consideration of the role that voices play in influencing speech perception.

Perceiving speaker identity from voice information is important for human social interactions. In adults, mechanisms involved in analyzing linguistic and speaker information appear to be partially dissociable (reviewed in Belin et al., 2004, 2011; Belin, 2006). Neuroimaging studies demonstrate that the brain can differentiate “who” from “what” is being said (Formisano et al., 2008), and cortical regions in the mid and anterior superior temporal gyrus respond selectively to human voices and are sensitive to speaker identity (Imaizumi et al., 1997; Belin et al., 2000; Nakamura et al., 2001; Belin and Zatorre, 2003; von Kriegstein et al., 2003). For pre-linguistic infants, extracting the identity of the speaker is likely just as important as deciphering meaning in speech. Even *in utero*, infants respond differently to the voice of their mother compared to a stranger (Kisilevsky et al., 2003), and behavioral and neuroimaging findings confirm that very young infants can indeed discriminate their mother’s voice from a stranger’s voice (DeCasper and Fifer, 1980; Dehaene-Lambertz et al., 2010), their father’s voice from other males (DeCasper and Prescott, 1984), and male from female voices (Jusczyk et al., 1992). Interestingly, the age at which infants become able to identify voices of individuals other than their mother’s remains unsettled (Brown, 1979; Hepper et al., 1993; Kisilevsky et al., 2003, 2009; Lee and Kisilevsky, 2014). In any case, these studies indicate that specialization for processing human voices appears to develop over infancy in conjunction with language experience (Grossmann et al., 2010; Vouloumanos et al., 2010; Johnson et al., 2011; Friendly et al., 2014; Schultz et al., 2014).

Humans are not the only species that can extract different types of information from conspecific vocalizations. The ability to distinguish and recognize individual conspecifics in the context of neighbor-stranger recognition has been found in numerous animals (including invertebrates) across the entire animal kingdom (see Tibbetts and Dale, 2007). For instance, Kentucky warblers and hooded warblers know both identity and location of each of their neighbors, and will respond aggressively to neighbor songs that are broadcasted at incorrect territorial boundaries (Godard, 1991; Godard and Wiley, 1995). Female great tits can discriminate between the highly similar songs of their mate and male neighbors (Blumenrath et al., 2007), and may eavesdrop on territorial mate-neighbor singing interactions in order to assess potential extra-pair partners (Otter et al., 1999). Vocal kin recognition has also been repeatedly demonstrated in various birds and mammals (e.g., Rendall et al., 1996; Holekamp et al., 1999; McComb et al., 2000; Illmann et al., 2002; Sharp et al., 2005; Janik et al., 2006; Akçay et al., 2014). Parent-offspring recognition by non-nesting penguins such as the king penguin offers a very strong case of vocal recognition because the chicks must accurately identify the calls of its parents within a crowded and noisy colony with almost no aid from visual or spatial cues. To identify parents, king penguin chicks pay attention to frequency modulations over time in conjunction with a beat analysis (Jouventin et al., 1999; Aubin et al., 2000). In contrast, chicks of nesting penguin species, such as Adelie penguin and gentoo penguin, that can use nesting site as an additional cue for parent identification will use simpler identification mechanisms that rely primarily on pitch and ignore frequency modulations (Jouventin and Aubin, 2002).

The difference between nesting and non-nesting penguins described above highlights the fact that individual recognition by auditory means may be achieved in many ways depending on ecological pressures and evolutionary history. For instance, birds can recognize a conspecific by the songs in his repertoire, by a particular variation in his song, or by his voice characteristics (Weary and Krebs, 1992). European starlings that each have a large song repertoire do not recognize other individual starlings by voice characteristics but rather by memorizing song motifs of different individuals (Gentner and Hulse, 1998; Gentner et al., 2000). On the other hand, great tits also possess a song repertoire but rely on voice characteristics to identify conspecific individuals (Weary and Krebs, 1992). In these experiments, recognition was assessed by training birds to discriminate songs from two individuals and testing their ability to discriminate other previously unheard songs from the same individuals (Weary and Krebs, 1992; Gentner and Hulse, 1998; Gentner et al., 2000). Animals are also capable of heterospecific vocal recognition of familiar individuals, as demonstrated in some monkey species that differentiated between the vocalizations of familiar and unfamiliar members of other monkey species (Candiotti et al., 2013). Observations like this suggests that animals may also be able to distinguish different humans based on their voice characteristics.

Identification of particular human individuals can be useful for animals because they can behave adaptively toward threatening and unthreatening humans by interrupting normal behavior or ignoring them, respectively. Visual discrimination and recognition of individual humans has been documented in several animals such as the magpie (*Pica pica;* Lee et al., 2011), mockingbird (*Mimus polyglottos;* Levey et al., 2009), crow (*Corvus brachyrynchos;* Marzluff et al., 2010, 2012), and octopus (*Enteroctopus dofleini*; Anderson et al., 2010). Some evidence also exists that animals raised in close contact with humans can distinguish between different human voices, which we have summarized in **Table 1**. In most of these studies, recognition was measured by preferential looking times when animals were presented with human faces and a voice that did or did not match one of the faces being shown. The design and interpretation of these studies are similar to those reported for infant studies (see Houston-Price and Nakai, 2004): longer looking times when faces and voices were mismatched were taken as an indication that subject’s expectations about the relationship between speaker face and speaker voice (i.e., speaker identity) were violated.

**Table 1:** List of studies that have tested animals’ discrimination of different human voices.

Reference	Species	Method	Comparison	Stimulus
Adachi et al. (2007)	Dogs	Face-voice matching	FvU	Animal’s name
Sliwa et al. (2011)	Rhesus macaques	Face-voice matching	FvF	Six standardized phrases *(e.g., “bonjour tout le monde”, “voila”)*
Lampe and Andre (2012)	Horses	Live person-voice matching	FvU	Standardized phrase *“Hey, [animal’s name], what are you doing there? Are you having a good day today? We have many riding lessons this week don’t we? The semester has started at JMU. You be a good boy/girl today!”*
Proops and McComb (2012)	Horses	Live person-voice matching	FvU FvF	Animal’s name
Wascher et al. (2012)	Crows	Playback	FvU	*“Hey”*
Saito and Shinozuka (2013)	Cats	Habituation–dishabituation	FvU	Animal’s name
Ratcliffe et al. (2014)	Dogs	Live person-voice matching	Male vs. female	Four standardized phrases *“Hey!”, “Come on then”, “Good dog!”, “What’s this?”*
McComb et al. (2014)	Elephants	Playback	Male vs. female Man vs. boy Masaai vs. Kamba	standardized sentence *“Look, look over there, a group of elephants is coming”*

*Note: FvU, familiar versus unfamiliar voice, FvF, familiar versus familiar voice.

Of the studies in **Table 1**, only Sliwa et al. (2011) and Proops and McComb (2012) have shown that animals can identify human individuals by their voices because the researchers tested discrimination among multiple familiar humans. This is critically important for proving individual identification, as discriminating familiar from unfamiliar individuals is recognition at the class-level, not the individual level (Tibbetts and Dale, 2007). In our opinion, however, even these two studies do not definitively prove human voice recognition because they used the animal’s name or phrases that the animal may have been frequently exposed to. This could lead animals to form an association between the specific phrase(s) and the speaker, such that they may not generalize their recognition to novel utterances by the speaker. Thus, animals’ performances in these studies may not necessarily reflect genuine recognition of different human speakers based on voice characteristics. We encourage future studies in human voice recognition to incorporate this method of training (i.e., initial discrimination between sounds from two familiar speakers and then testing their ability to recognize different sounds from the same speakers) because it eliminates class-level recognition and learned associations between speaker and specific phrases as confounding factors. Alternatively, researchers may also consider using a habituation-dishabituation paradigm, where subjects are habituated to various speech sounds from one speaker and then tested on whether they dishabituate to speech sounds of a different speaker (e.g., Johnson et al., 2011).

Another fundamental component that these studies do not address is what auditory cues animals may be using to discriminate different human voices, and whether they use the same cues to identify conspecific calls. The question of which cues are used to identify speakers is highly relevant to humans as well, as there is currently no consensus on this matter (see Creel and Bregman, 2011). Some studies have found that adults use pitch and/or formants to identify speakers (e.g., Remez et al., 1987; Fellowes et al., 1997; Baumann and Belin, 2010), whereas others have suggested that these spectral cues are important for gender determination while temporal patterns are used for individual identification (Fellowes et al., 1997). Furthermore, not all listeners use the same cues to distinguish voices, and different cues may be more important for distinguishing particular voices (Kreiman et al., 1992).

We are not aware of any studies that investigate what acoustic cues animals may be using to discriminate or identify human voices, although this is surely a question that merits rigorous investigation. Work by Zoloth et al. (1979) suggests that conspecifics and heterospecifics may attend to different cues, as Japanese macaques used peak of a frequency inflection while other monkey species used initial pitch to discriminate between coos of other Japanese macaques. But if animals use the same cues for recognition of conspecific vocalization as heterospecific vocalization, then we expect that only some species will use voice characteristics for individual recognition. That is, following the example described above, we would expect great tits but not European starlings to recognize different human voices because great tits discriminate conspecifics based on voice characteristics while starlings discriminate conspecifics based on song motifs (Weary and Krebs, 1992; Gentner and Hulse, 1998; Gentner et al., 2000). Even then, great tits may rely on different parameters than human listeners, as the song parameters that show significant variation between individual great tits are the number of phrases in a song, duration of the first phase minus the last phase, maximum frequency, and pitch (Weary, 1990; Weary et al., 1990). The acoustic similarities between human speech and species-specific vocalizations may also determine which cues are used. For example, zebra finches distance calls have harmonic structures that resemble vowel formants in human speech (Dooling et al., 1995). This similarity may explain why zebra finches perform similarly to humans during discrimination of speech sounds (Dooling et al., 1995; Ohms et al., 2012), and why neurons in a secondary auditory area of the zebra finch brain respond more strongly to human speech and species-specific calls than calls of other songbirds that are acoustically less similar (Chew et al., 1996).

Findings from animal studies are valuable for understanding the extent to which human voices can be differentiated and recognized without the need for human voice-specific neural networks (Leaver and Rauschecker, 2010; Belin et al., 2011). However, research on human voice processing in animals to date is scarce, and of the studies that do exist, the ability to recognize voices (defined as discriminating between different familiar voices, as opposed to discriminating between familiar and unfamiliar voices) has yet to be compellingly demonstrated. An understanding of the differences and similarities of acoustic cues and mechanisms used for acoustic recognition of conspecifics and heterospecifics is also currently severely lacking. We strongly believe that these issues must be addressed in future studies if we are to discover parallels between voice processing in humans and animals, and consequently shed light onto the evolution of human voice perception.

## SPEAKER NORMALIZATION

The counterpart of being able to extract information about speaker identity is being able to handle acoustic variations of the same utterance caused by speaker differences. The acoustic realizations of phonemes and words can vary tremendously between speakers, due to physical, contextual, environmental, and sociolinguistic factors (i.e., age and gender differences in vocal tract size and shape, coarticulation, background noise, and accents). Consequently, speaker normalization refers to our ability to recognize phonologically identical utterances despite high acoustic variability across speakers (Johnson, 2005). A compelling example of the immense variability in the speech signal resulting from differences between speakers is in vowel production. Vowels are reliably distinguished by the first and second formant frequencies (F1 and F2); however, F1 and F2 values of vowels produced by different speakers (and especially different genders) are highly variable within a vowel category and greatly overlap between categories, to the extent that the acoustic distance within a vowel category can be just as large as the acoustic distance between vowel categories (Potter and Steinberg, 1950; Peterson and Barney, 1952; Hillenbrand et al., 1995). Consequently, studies that seek to understand our impressive ability to normalize vowels despite this intensive overlap (Peterson and Barney, 1952; Strange et al., 1976, 1983; Assmann et al., 1982) can provide convincing evidence of what processes contribute to speaker normalization.

Researchers have not yet reached a consensus on how vowel normalization in humans is achieved (reviewed in Nearey, 1989; Johnson et al., 1999; Adank, 2003; Johnson, 2005). Some argue that normalization occurs via low-level auditory perceptual processes, by computation of particular formant ratios that allow vowel categories to be distinctively represented in discrete regions in acoustic space (Potter and Steinberg, 1950; Syrdal and Gopal, 1986; Miller, 1989), or by using F0 or F3 values (i.e., fundamental frequency and third formant frequency) that are correlated with vocal tract length to disambiguate ambiguous F1 and F2 values (e.g., Ladefoged and Broadbent, 1957; Fujisaki and Kawashima, 1968; Wakita, 1977; Nearey, 1989). A recent paper suggests that humans normalize for speaker differences by computing ratios between F1 and F2 in relation to F3. Specifically, Monahan and Idsardi (2010) showed that transforming F1 and F2 values into F1/F3 and F2/F3 ratios effectively eliminated variation between speakers in a corpus of American English vowels from Hillenbrand et al. (1995). They also provided neurophysiological evidence that the human brain is sensitive to F1/F3 ratios, complementing prior work showing that the brain is sensitive to F2/F3 ratios (Monahan and Idsardi, 2010). Alternatively, others argue that listeners form rich perceptual representations of speaker identity by incorporating vocal tract length with other learned factors such as familiarity or socio-cultural expectations; these abstract speaker representations subsequently influence vowel normalization (i.e., “talker normalization”; Johnson, 1990; Johnson et al., 1999).

Both types of views have merits and drawbacks, and they have also both received empirical support. The auditory perceptual approaches can account for how vowel normalization occurs with limited linguistic capabilities and familiarity with novel speakers (Monahan and Idsardi, 2010). Behavioral and neurophysiological studies indicate that humans normalize speaker differences in vowels without linguistic comprehension or attention. In adults, extraction and processing of vowel formants takes place at a subcortical and pre-attentional level (von Kriegstein et al., 2006; Monahan and Idsardi, 2010; Tuomainen et al., 2013), and that even pre-linguistic infants can categorize vowels of different speakers and genders (Kuhl, 1983). On the other hand, talker normalization approaches explain how listeners can learn speaker-specific and language-specific patterns of speech (Johnson et al., 1999). Several findings support the idea that learning of non-acoustic speaker-related variables is indeed involved in accommodating for speaker differences in phonetic realizations (Samuel and Kraljic, 2009). For instance, expectations of speaker gender can alter phoneme boundaries (Johnson et al., 1999), single speaker and familiar speakers conditions yield better vowel identification (Strange et al., 1976, 1983; Assmann et al., 1982; Mullennix et al., 1989; Nygaard and Pisoni, 1998), and infants and adults can rapidly adapt to accented speech (Clarke and Garrett, 2004; Bradlow and Bent, 2008; White and Aslin, 2011; Cristia et al., 2012; van Heugten and Johnson, 2014)

Comparative studies in animals represent one way to address whether speaker normalization mechanisms are unique to humans. Although normalization is necessary for word recognition, normalization also occurs for phonetic segments. Indeed, normalization has been demonstrated in 6 month-old infants before they acquire words, and at the level of vowels (Kuhl, 1983). That is, non-verbal infants can normalize speech even before they have learned words. Even for the few words that 6–9 month-old infants do seem to recognize (Bergelson and Swingley, 2012), normalization may not have been required for recognition, since the words were spoken by a familiar speaker (i.e., a parent). Therefore, it is important to discern what perceptual processes infants use to normalize speech, and whether these mechanisms are rudimentary processes that are available to infants because they are species-shared or non-speech related perceptual mechanisms. Unfortunately, only a handful of animal studies have considered speaker variability as a factor, even though the ability to account for speaker-dependent variation is fundamental for speech perception. A possible reason for lack of interest in how animals handle speaker-dependent variation in speech may have to do with skepticism regarding what, if any, benefits this investigation would yield. In other words, as suggested by Trout (2001), what reason would an animal have to normalize speaker variation in speech? What advantages would it confer to the animal? And even if animals do appear to normalize speech, are they applying similar mechanisms to humans? We argue that studying speaker normalization in non-human animals is ecologically valid because animals also have to deal with signal variability when recognizing vocalizations made by other individuals of the same species (conspecifics) and other individuals of a different species (heterospecifics). On the other hand, whether animals and humans attend to the same cues and have shared mechanisms for normalizing human speech is an open and exciting question that is yet to be answered.

Signal variability in animal vocalizations can be caused by inter-individual differences, such as physical size (Fitch, 1997, 1999; Growcott et al., 2011), intra-individual differences, such as affective state (e.g., stress; Perez et al., 2012), and a combination of inter-and intra-individual differences such as fluctuations in endocrine state (Galeotti et al., 1997; Soma et al., 2002). Yet animals must still be able to acquire information about the type of vocalization (such as calls indicating predator versus food source) and in some contexts, the identity of the signaler. Thus, in parallel to how humans can extract linguistic information despite speaker-dependent variation in the speech signal, animals can also categorize vocalizations of conspecific as well as heterospecific vocalizations despite individual variation in the signal. Indeed, responding to hetereospecific signals is widespread in many animals because of the advantages it confers to the receiver (Seppänen et al., 2007). For example, red-breasted nuthatches (*Sitta canadensis*) are sensitive to variations in black-capped chickadee (*Poecile atricapillus*) mobbing calls that encode information about the size and degree of threat of predators (Templeton and Greene, 2007), avian brood parasites may eavesdrop on sexual signals of host species to assess parental quality (Parejo and Avilés, 2007), and hornbills can respond to differentially to alarm calls of Diana monkeys that signal significant and non-significant threats for the hornbills (Rainey et al., 2004).

The red-breasted nuthatches’ extraction of information from the black-capped chickadee *chick-a-dee* call is particularly compelling because of the highly sophisticated nature of this vocalization, both at a contextual and an acoustic level. In addition to signaling predators, the *chick-a-dee* call is used in other contexts, such as to maintain group cohesion and coordinate group movements (Ficken et al., 1978). Significant individual differences in the *chick-a-dee* call can occur in every 100-Hz interval between 500 and 7000 Hz, with greater variation between individuals than within individuals of the same group in various temporal and spectral parameters (Mammen and Nowicki, 1981). Acoustically, the call is made of four note types (A, B, C, D) sung in a fixed sequence, but note types can be repeated or omitted to create 100s of variations such as ACCDD, AABBCD, or ADDDD (Hailman et al., 1985). Black-capped chickadees can also modify spectral components of the D note so that this note converges amongst members of the same group *(*Nowicki, 1989*).*

Importantly, the same variations in syntax (number of D notes), temporal and spectral characteristics (duration and interval between D notes, frequency overtone spacing and bandwidth) are also used to convey information about predator threat (Templeton et al., 2005). That is, the same acoustic properties that encode predator threat also vary depending on the individual. Yet despite the apparent overlap between acoustic parameters that signal individual/group identity and predator threat, nuthatches are still able to obtain information relevant for their behaviors. Thus, this constitutes a naturally occurring example of normalization of heterospecific vocal signals by a non-human animal (i.e., disregarding irrelevant variation caused by individual differences in the vocalizations of another species). In this light, the idea that non-human animals can account for speaker differences in human vocalizations seems quite plausible, and in the following sections we review studies that suggest that speaker normalization of speech is not a uniquely human ability.

The ability to normalize speaker differences in naturally spoken vowels or correlates of gender differences in synthetic vowels (i.e., F0, which is generally higher in female than male voices and is utilized in perception of voice gender by humans; Hillenbrand et al., 1995; Mullennix et al., 1995; Lavner et al., 2000) has been tested in cats, rhesus monkeys, dogs, chimpanzees, chinchillas, budgerigars, rats, and ferrets (Dewson, 1964; Dewson et al., 1969; Baru, 1975; Burdick and Miller, 1975; Kojima and Kiritani, 1989; Dooling and Brown, 1990; Eriksson and Villa, 2006; Bizley et al., 2013). All of these studies reported that animals were able to differentiate stimuli based on vowel category and ignore speaker-dependent variation. However, we argue that none of these studies robustly demonstrate speaker normalization. This is because the experiments on cats, dogs, rhesus monkeys, chimpanzees, chinchillas, and budgerigars tested discrimination of the vowels /i/, /u/, and or /a/ (Dewson, 1964; Dewson et al., 1969; Baru, 1975; Burdick and Miller, 1975; Kojima and Kiritani, 1989). As these vowels occupy distant acoustic spaces, the variability between these vowel categories is likely larger than the variability between speakers. Consequently, a stronger test for vowel normalization would use vowel categories where speaker differences and vowel category differences have relatively equal variation. This was addressed by Dooling and Brown (1990), who found that budgerigars could discriminate between the psychoacoustically closer vowels /i/ and /ε/ produced by different male speakers. However, they did not examine whether budgerigars could also discriminate these vowels produced by different female speakers, so we cannot be sure that budgerigars can in fact normalize these vowels because much of the acoustic overlap between vowel categories are due to gender differences (see Peterson and Barney, 1952). Lastly, the experiments in rats, ferrets, and chimpanzees used synthetic vowels that differed only in one acoustic parameter (Kojima and Kiritani, 1989; Eriksson and Villa, 2006; Bizley et al., 2013). This is problematic for conclusions about speaker normalization because voices differ in various dimensions, and no single acoustic cue has been found that reliably predicts speaker characteristics that can be used to normalize vowels.

As far as we know, Ohms et al. (2010) are the only researchers that have clearly demonstrated that an animal can normalize vowels of different speakers. Specifically, Ohms et al. (2010) found that zebra finches can generalize their discrimination of two words that differ only in vowels (wɪt and wεt) to multiple, different male or female speakers after being trained to discriminate these words from a single speaker of the same sex. Their results show that birds are indeed learning something about the phonemic differences between wɪt and wεt, and are able to apply these criteria to unfamiliar speakers while ignoring speaker-dependent differences in production of these words. Notably, zebra finches could also recognize the same word produced by male and female speakers after learning the word from a set of speakers of the other sex (Ohms et al., 2010; see also ten Cate, 2014). Remarkably, this ability seems lacking in human infants (Houston and Jusczyk, 2000). A logical follow up of these results would be a test for normalization of isolated vowels – an ability demonstrated by human adults (Strange et al., 1976, 1983; Assmann et al., 1982). This is because the birds in Ohms et al. (2010) could have been using other acoustic cues instead of vowel differences during generalization, such as formant transitions between consonant and vowel. Recently, a study by Engineer et al. (2013) reported that rats too could normalize speaker variation in consonants. Rats could learn to discriminate between the words *dad* and *tad* produced by a female speaker and generalize this discrimination to novel male and female speakers. With the same stimuli, rats could also learn to distinguish between the word *dad* spoken by a female speaker and the same word that was pitch-shifted down by one octave, and subsequently generalize this discrimination to novel male and female voices (Engineer et al., 2013). This shows that, like humans, animals can extract different types of information from speech (see section on speaker voice recognition).

Griebel and Oller (2012) provide another interesting case that may potentially reflect normalization of speaker differences by a Yorkshire terrier (Bailey). They found that Bailey could correctly retrieve 13 out of 16 familiar toys when verbally requested by a female experimenter with a German accent and a male experimenter with a western American English (California) accent, even though Bailey’s owner was female with a southern American English (Tennessee) accent. Bailey’s accurate performance with these accented voices was not due to training or familiarity, as the experimenters had never previously requested the toys from Bailey. Again, we do not know which acoustic parameters Bailey was using to recognize familiar words spoken by unfamiliar speakers. The authors note that toy names often consisted of “two or more words that included intonation and alliteration or assonance cues that may have made them easier to remember and discriminate” (Griebel and Oller, 2012). This suggests that Bailey may not have needed to normalize speaker differences in vowel production in order to recognize the words, but possibly relied on prosodic cues – another feature in speech that tamarins (Ramus et al., 2000), rats (Toro et al., 2003), Java sparrows (Naoi et al., 2012), and zebra finches (Spierings and ten Cate, 2014) are sensitive to.

Other studies on word discrimination in dogs have been conducted, but do not offer clear evidence for speaker normalization. This is because these studies do not control for non-verbal cues from the trainer that dogs could rely on to perform correct actions (see Mills, 2005), or they test dogs’ ability to retrieve objects from the verbal commands of a single familiar trainer (Warden and Warner, 1928; Kaminski et al., 2004; Fukuzawa et al., 2005). Another study did not explicitly state in the methods whether commands were consistently given by a single trainer or by multiple trainers (Ramos and Ades, 2012). In a similar vein, Warfield et al. (1966) showed that cats could discriminate the words “bat” and “cat,” but they did not test whether discrimination was affected if word tokens were produced by multiple speakers.

Responding to categories of heterospecific communication signals is commonly found in the animal kingdom, and though few studies provide direct evidence, there is some indication that animals may be able to normalize speaker differences in human speech. Yet replication and extension of positive findings such as those by Ohms et al. (2010) and Engineer et al. (2013) are compulsory. In particular, we point out that the ability to categorize speech sounds from different speakers and disregard non-essential information is not an absolute demonstration of normalization, which is why researchers must test whether animals and humans apply the same normalization mechanisms. For example, do both humans and animals successfully categorize vowels of multiple speakers by computing formant ratios as proposed by Monahan and Idsardi (2010), or by using other normalization algorithms that have been previously proposed (reviewed in Escudero and Bion, 2007). Such tests would be invaluable in verifying whether similar behaviors exhibited by humans and animals are mediated by the same or different mechanisms.

## CONCLUSION

In this review we have discussed the current state of animal research in three aspects of speech and voice perception. We have presented arguments and evidence that caution against premature conclusions about whether asymmetries in vowel perception reflect an innate and uniquely human bias that is present in inexperienced language learners and not attributable to general properties of the vertebrate or mammalian auditory system. We have noted that there is a lack of definitive evidence for animal recognition of individual human voices in the literature, and we have suggested ways to improve experimental designs that will more assuredly test whether animals can use voice characteristics to discern different humans. Lastly, we infer with reservation from two recent experiments that animals may be able to normalize speaker differences. Consequently, we strongly encourage researchers to conduct more carefully designed and rigorously controlled experiments to validate the human-specific claims of asymmetries in vowel perception, voice perception, and speaker normalization that we have described. Studies that identify what acoustic cues animals rely on to perform either individual voice perception or speaker normalization are also seriously needed as they are invaluable for our understanding of how these behaviors are accomplished.

With accumulating empirical results, we can sooner reach the stage where findings from these three areas can be synthesized to tackle broader questions in speech perception. An example would be whether and at what level of processing speaker identification and speaker normalization mechanisms interact during speech perception. Some researchers believe that in humans the analysis of linguistic information and speaker identity during speech perception may be segregated into dissociable but interacting neural pathways (although the stage at which the integration of these two streams occurs is undetermined; Belin et al., 2004). Comparative research on whether animals also analyze conspecific vocalizations and/or human speech for communicative content and signaler identity separately would reveal whether or not this compartmentalization occurred as a distinctive human adaptation that enables us to map overlapping and highly variable acoustic information onto correct phonetic categories while simultaneously processing speaker identity related cues.

We have emphasized throughout this paper that addressing these topics in animals is neither insignificant nor extraneous because many social animals encounter similar challenges to those humans face when discriminating similar-sounding vocalizations/phonemes, determining signaler/speaker characteristics from vocalizations/speech, and resolving between-individual variation in order to perceive the content of vocalizations/speech. It is our hope that this review will have cogently demonstrated that expanding our view to include how animals perceive speech can offer valuable insights for more thorough conceptualization of the specificity, simplicity (or complexity), and specialization of human speech perception mechanisms.

## Conflict of Interest Statement

The authors declare that the research was conducted in the absence of any commercial or financial relationships that could be construed as a potential conflict of interest.
